# Supplementary material for: Ultra-Processed Food Consumption Is Associated with an Increased Risk of Abdominal Obesity in Adults: A Cross-Sectional Study in Shanghai
Source: Foods. 2025 Nov 18;14(22):3955. doi: 10.3390/foods14223955 (PMC12651846; doi:10.3390/foods14223955)
Supplement: Supplementary file 1 [file foods-14-03955-s001.zip › Supplementary Materials.pdf]

**Table S1.** Mean and standard deviations (SDs) of transformed Energy and main nutrients intake

| | non/low consumption group (n=949) | medium consumption group (n=945) | high consumption group (n=948) | F | *p* |
|---|---|---|---|---|---|
| **Energy, kcal/d** | -0.079(1.041) | 0.007(1.011) | 0.071(0.939) | 10.650 | 0.001 |
| **Protein, g/d** | -0.074(1.058) | 0.021(1.014) | 0.052(0.918) | 7.595 | 0.006 |
| **Fat, g/d** | -0.078(1.049) | 0.048(1.016) | 0.030(0.926) | 5.540 | 0.019 |
| **Carbohydrate, g/d** | -0.066(1.055) | 0.063(0.995) | 0.002(0.942) | 2.283 | 0.131 |
| **Cholesterol, mg/d** | -0.063(1.066) | -0.033(0.975) | 0.096(0.947) | 12.005 | 0.001 |
| **Dietary fiber, g/d** | -0.006(1.048) | 0.017(0.976) | -0.011(0.973) | 0.013 | 0.911 |
| **Calcium, mg/d** | -0.128(1.023) | 0.061(0.982) | 0.067(0.982) | 18.141 | <0.001 |
| **Iron, mg/d** | 0.067(0.971) | 0.026(0.996) | -0.094(1.024) | 12.434 | <0.001 |
| **Phosphorus, mg/d** | -0.052(1.048) | 0.047(0.991) | 0.005(0.955) | 1.571 | 0.210 |
| **Potassium, mg/d** | -0.044(1.034) | 0.033(0.997) | 0.012(0.966) | 1.494 | 0.222 |
| **Sodium, mg/d** | -0.012(1.041) | 0.035(0.969) | -0.023(0.987) | 0.051 | 0.821 |
| **Vitamin A, mg/d** | 0.004(1.051) | 0.037(0.954) | -0.042(0.990) | 1.057 | 0.304 |
| **Carotene, mg/d** | 0.134(1.019) | -0.003(0.965) | -0.131(0.997) | 33.609 | <0.001 |
| **Thiamine, mg/d** | 0.046(1.061) | -0.001(0.980) | -0.046(0.954) | 4.087 | 0.053 |
| **Riboflavin, mg/d** | -0.089(1.039) | 0.068(0.984) | 0.020(0.969) | 5.636 | 0.018 |
| **Niacin, mg/d** | 0.026(1.010) | 0.016(0.983) | -0.043(1.006) | 2.317 | 0.128 |
| **Vitamin C, mg/d** | 0.050(1.041) | 0.035(0.958) | -0.085(0.993) | 8.701 | 0.003 |
| **Vitamin E, mg/d** | 0.055(1.040) | 0.055(0.982) | -0.111(0.967) | 13.174 | 0.001 |
| **Folic acid, mg/d** | 0.071(1.051) | -0.004(0.962) | -0.036(0.901) | 5.844 | 0.016 |
| **Overall** | | | | 16.423 | <0.001 |

One-way multivariate analysis of variance revealed significant differences between the groups (Wilks' Lambda=0.8998, F=16.422, $p<0.001$) in table S1. These differences were statistically significant in energy(F=10.650,$p$=0.001), protein(F=7.594,$p$=0.006), fat(F=5.540,$p$=0.018), cholesterol(F=12.004,$p$=0.001), calcium(F=18.141,$p<0.001$), iron(F=12.434,$p$=0.001), carotene(F=33.608,$p<0.001$), thiamine(F=4.087,$p$=0.043), riboflavin(F= 5.635,$p$=0.018), vitamin C(F=8.701, $p$=0.003), vitamin E(F=13.174,$p$=0.001) and Folic acid(F=5.844,$p$=0.016).
